# Attenuation of High-Fat Diet-Induced Rat Liver Oxidative Stress and Steatosis by Combined Hydroxytyrosol- (HT-) Eicosapentaenoic Acid Supplementation Mainly Relies on HT

**DOI:** 10.1155/2018/5109503

**Published:** 2018-07-02

**Authors:** Francisca Echeverría, Rodrigo Valenzuela, Andrés Bustamante, Daniela Álvarez, Macarena Ortiz, Sandra A. Soto-Alarcon, Patricio Muñoz, Alicia Corbari, Luis A. Videla

**Affiliations:** ^1^Department of Nutrition, Faculty of Medicine, University of Chile, Santiago, Chile; ^2^Nutrition and Dietetics School, Faculty of Health Sciences, Catholic University of Maule, Curicó, Chile; ^3^Molecular and Clinical Pharmacology Program, Institute of Biomedical Sciences, Faculty of Medicine, University of Chile, Santiago, Chile

## Abstract

Pharmacological therapy for nonalcoholic fatty liver disease (NAFLD) is not approved at the present time. For this purpose, the effect of combined eicosapentaenoic acid (EPA; 50 mg/kg/day) modulating hepatic lipid metabolism and hydroxytyrosol (HT; 5 mg/kg/day) exerting antioxidant actions was evaluated on hepatic steatosis and oxidative stress induced by a high-fat diet (HFD; 60% fat, 20% protein, and 20% carbohydrates) compared to a control diet (CD; 10% fat, 20% protein, and 70% carbohydrates) in mice fed for 12 weeks. HFD-induced liver steatosis (i) was reduced by 32% by EPA, without changes in oxidative stress-related parameters and mild recovery of Nrf2 functioning affording antioxidation and (ii) was decreased by 42% by HT, concomitantly with total regain of the glutathione status diminished by HFD, 42% to 59% recovery of lipid peroxidation and protein oxidation enhanced by HFD, and regain of Nrf2 functioning, whereas (iii) combined EPA + HT supplementation elicited 74% reduction in liver steatosis, with total recovery of the antioxidant potential in a similar manner than HT. It is concluded that combined HT + EPA drastically decreases NAFLD development, an effect that shows additivity in HT and EPA effects that mainly relies on HT, strengthening the impact of oxidative stress as a central mechanism underlying liver steatosis in obesity.

## 1. Introduction

Oxidative stress is a disequilibrium condition in which the cellular redox balance is shifted towards a more oxidizing status that may trigger adaptation of cellular functions [1]. Depending on the antioxidant level of different cell types, the concentration of reactive oxygen species (ROS) achieved, and the duration of the exposure, oxidative stress may trigger beneficial responses under mild conditions and potentially harmful ones beneath severe situations, as a typically hormetic phenomenon [2]. In the latter case, oxidative stress development plays a role in the pathogenesis of several liver diseases, including alcoholic liver disease, haemochromatosis, Wilson's disease, chronic hepatitis C, and nonalcoholic fatty liver disease (NAFLD) [3]. NAFLD is characterized by excess triglyceride (TG) deposition in the hepatocyte followed by development of inflammatory (nonalcoholic steatohepatitis (NASH)) and fibrogenic responses [4] as shown in patients with obesity and insulin resistance [5–7]. Liver steatosis is also observed in rodents subjected to high-fat diets (HFDs) [8], which are considered adequate experimental models to understand the underlying mechanisms that may support dietary and/or nutritional interventions preventing or treating NAFLD [9, 10]. In addition to liver steatosis and oxidative stress, HFDs containing 45% to 75% of their calories as fat for 12 to 16 weeks induce overweight, insulin resistance, a proinflammatory status, apoptosis, and n-3 long-chain polyunsaturated fatty acid (n-3 LCPUFA) depletion [8–11], diets that are characterized by being sufficient in macro- and micronutrients [12, 13].

Regardless of the high prevalence and increasing incidence of adult and paediatric NAFLD, no pharmacological therapy for NAFLD or NASH is approved at the present time, weight loss and exercise being the mainstay of treatment [14]. Considering that NAFLD is a multifactorial entity, it has been considered that combined therapies may achieve higher rates of responses and improved outcomes than monotherapies [14–16]. Supporting this contention, combined therapies using (i) thyroid hormone- (T_3_-) docosahexaenoic acid (DHA) prevent ischemia-reperfusion-induced liver inflammatory injury [17] and (ii) DHA-extra virgin olive oil (EVOO) attenuates HFD-dependent hepatic steatosis [18], whereas (iii) combination drug treatments have been proposed in the case of uncontrolled hypertension [19] and (iv) high-potency statins combined with ezetimibe or pioglitazone were recommended for the resolution of NAFLD or NASH [20]. Based on the hepatoprotective effects of the EVOO component hydroxytyrosol (HT) [21, 22] and the n-3 LCPUFA eicosapentaenoic acid (EPA) [23], we hypothesized that the combined supplementation with EPA and HT could alter HFD-induced biochemical changes associated with steatosis. For this purpose, general metabolic parameters were measured concomitantly with the fatty acid (FA) composition and degree of steatosis, the oxidative stress status, and the activity of antioxidant enzymes controlled by the redox-sensitive transcription factor nuclear factor erythroid 2-related factor 2 (Nrf2) in the liver of mice subjected to HFD (60% of the total calories as fat for 12 weeks).

## 2. Methods

### 2.1. Animals and Diet Supplementation

Weaning male C57BL/6J mice weighing 12–14 g (Bioterio Central, ICBM, Faculty of Medicine, University of Chile) were randomly assigned to each experimental group (*n* = 8 per experimental group) and were allowed free access to control diet (CD) or HFD. The CD composition (expressed as % total calories) was 10% fat, 20% protein, and 70% carbohydrate, with a caloric value of 3.85 kcal/g, and HFD composition was 60% fat, 20% protein, and 20% carbohydrate, with a caloric value of 5.24 kcal/g (Rodent Diet, product data D12450B and D12492, Research Diet Inc., USA). FA composition of CD and HFD was previously described [9]. Animals received water ad libitum and were housed on a 12 h light/dark cycle from days 1 to 84 (12 weeks). EPA, isolated from fish oil (Golden Omega S.A., Chile) as TAG (50% EPA, 5% DHA, and 5% of other n-3 FAs; 15% saturated fatty acid (SFA) (principally palmitic acid), and 25% MUFA (principally oleic acid)), was administered at 50 mg/kg/day dosage. HT (elaVida™, DSM Nutritional Products Company, Netherlands) was given at doses of 5 mg/kg/day, and control groups received isovolumetric amounts of saline orally, conforming eight experimental groups, namely, (a) CD (control), (b) CD + EPA, (c) CD + HT, (d) CD + EPA + HT, (e) HFD, (f) HFD + EPA, (g) HFD + HT, and (h) HFD + EPA + HT. The doses of EPA or HT used in this study were used according to previous research; namely, EPA (50 mg/kg) represents 50% of the dose of EPA + DHA used by Valenzuela et al. [8], whereas HT at 5 mg/kg exhibits protective effects against HFD [24]. Weekly controls of body weight and diet intake were performed through the whole period, and at the end of the 12th week the animals were fasted (6–8 h) and anesthetized with isoflurane (Lunan Baxter Pharmaceuticals Co. Ltd., Shandong, China), and blood samples were obtained by cardiac puncture for the determination of serum aspartate transaminase (AST) and alanine transaminase (ALT), together with the oxidative stress status of the liver. Liver samples were either frozen in liquid nitrogen for determination of FA composition or fixed in phosphate-buffered formalin, embedded in paraffin, stained with haematoxylin-eosin, and analysed by optical microscopy in a blind fashion describing the presence of steatosis and inflammation, both graded as absent, mild, moderated, and severe [25].

### 2.2. Ethics

All animal procedures in this study were in strict adherence to the Guide for the Care and Use of Laboratory Animals (National Academy of Sciences, NIH Publication 6–23, revised 1985) and were approved by the Bioethics Committee for Research in Animals, Faculty of Medicine, University of Chile (CBA protocol 0580 FMUCH).

### 2.3. Biochemical Analyses and Oxidative Stress Markers

Serum AST and ALT activities (U L^−1^) were measured using specific diagnostic kits (bioMérieux SA, Marcy l'Etoile, France). Total fat content in liver (mg/g) was evaluated according to Bligh and Dyer [26], and triacylglycerol (TAG) content (mg g^−1^) was measured using specific kits according to the manufacturer's instructions (Cayman Chemical Company, Michigan, USA). Livers from anesthetized animals were perfused in situ with a cold solution containing 150 mM KCl and 5 mM Tris (pH 7.4) to remove blood for protein carbonylation and glutathione assessments. Protein carbonyl concentration was determined by a fluorometric assay (Cayman Chemical Company, Michigan, USA) after adjusting the total protein concentration to 7.5 mg mL^−1^ per sample. Reduced glutathione (GSH) and glutathione disulphide (GSSG) contents were assessed with an enzymatic recycling method [27]. The antioxidant capacity of serum, serum and liver thiobarbituric acid reactive substances (TBARS), and hepatic F-8 isoprostanes were determined by colorimetric assays (Cayman Chemical Company, Michigan, USA).

### 2.4. Determination of Liver Antioxidant Enzyme Activities

The liver activity of CAT was measured according to the method of Lück [28]. Assessment of SOD activity was carried out with a commercial assay kit (Cayman Chemical Company; Michigan, USA) according to the manufacturer's instructions. GPX activity was determined using the method described by Paglia and Valentine [29]. GR activity was determined according to Horn [30], NADPH-quinone oxidoreductase 1 (NQO1) activity was measured according to the method of Ernster et al. [31], glutathione-S-transferase (GST) activity was determined according to the method described by Habig et al. [32], and *γ*-glutamyl transpeptidase (GGT) activity was determined following the method of Satomura et al. [33]. To develop the experimental specific conditions for evaluating the liver activity of these enzymes, we followed the methods previously published by Rincón-Cervera et al. [34] and Valenzuela et al. [24].

### 2.5. Gene Expression Assays

Total RNA was isolated from liver samples using TRIzol (Invitrogen, Paisley, UK), according to the supplier's protocols. Purified RNA (2 *μ*g) was then treated with DNase (DNA-free kit; Ambion, Austin, TX, USA) and used to generate first-strand cDNA with M-MLV Reverse Transcriptase (Invitrogen, Paisley, UK), utilizing random hexamers (Invitrogen, Paisley, UK) and dNTP mix (Bioline, London, UK), according to the manufacturer's protocol. The resultant cDNA was amplified with specific primers for mice in a total volume of 10 *μ*L.  Table 1 depicts the gene-specific primer sequences used in the study. Primer optimization and real-time quantitative PCR were performed according to Rincón-Cervera et al. [34].

### 2.6. Assessment of Liver DNA-Binding Activity of Nrf2

Nuclear extracts from liver tissue (left lobe) were obtained using a commercial extraction kit (Cayman Chemical Company, Michigan, USA). Nrf2 DNA-binding activity was assessed with commercial ELISA kits (Cayman Chemical Company, Michigan, USA), according to the manufacturer's instructions. Values were expressed as percentage of Nrf2 DNA binding with respect to a positive control provided by the ELISA kit.

### 2.7. FA Profile

Quantitative extraction of total lipids from liver was carried out according to Bligh and Dyer [26]. Liver samples were homogenized in ice-cold chloroform/methanol (2 : 1 *v*/*v*) containing 0.01% butylated hydroxytoluene in an Ultra-Turrax homogenizer (Janke & Kunkel, Stufen, Germany). Total lipids from liver samples were extracted with chloroform/methanol (2 : 1 *v*/*v*). Fatty acid methyl esters (FAMEs) from total liver fat were prepared as previously described [34] and analysed according to Valenzuela et al. [9].

### 2.8. Statistical Analysis

Statistical analysis was performed with GraphPad Prism version 6.1 (GraphPad Software, San Diego, CA, USA). Values shown represent the mean ± SEM for the number of separate experiments indicated. Two-way ANOVA and Bonferroni's post hoc test assessed the statistical significance of differences between mean values, with *p* < 0.05 being considered significant. Pearson's coefficient was used to assess associations between variables.

## 3. Results

### 3.1. General Parameters, Food and Energy Intake, and Liver Function-Related Indexes

Mice in the different experimental groups exhibiting comparable initial body weights showed 75% to 95% increases in their final body weights when given CD, with an average body weight gain of 12.4 ± 0.7 g (*n* = 64) that was enhanced by 85% by HFD alone (group e; Table 2). EPA or EPA + HT supplementation in HFD mice generated a significant reduction in body weight gain, 20% and 33%, respectively, whereas HT supplementation was not significant in this parameter (Table 2). In all experimental groups, dietary intake was comparable, but energy consumption was higher in mice subjected to HFD without or with EPA, HT, and EPA + HT supplementation over values in the respective CD groups. Under these conditions, serum AST and ALT levels were comparable in all groups, whereas liver weight in the HFD + EPA + HT group was reduced by 18% (*p* < 0.05) compared to CD values. Furthermore, hepatic total fat was comparable in mice subjected to CD without or with supplementations, similarly to liver TG levels; however, hepatic fat increased by 193% due to HFD, and liver TGs were elevated by 210% over CD values (*p* < 0.05). The latter two parameters in HFD mice were not altered by EPA or HT, but decreased by 75% and 63% (*p* < 0.05) by EPA + HT supplementation, respectively (Table 2).

### 3.2. Liver Morphological Characteristics

**Figure 1:**
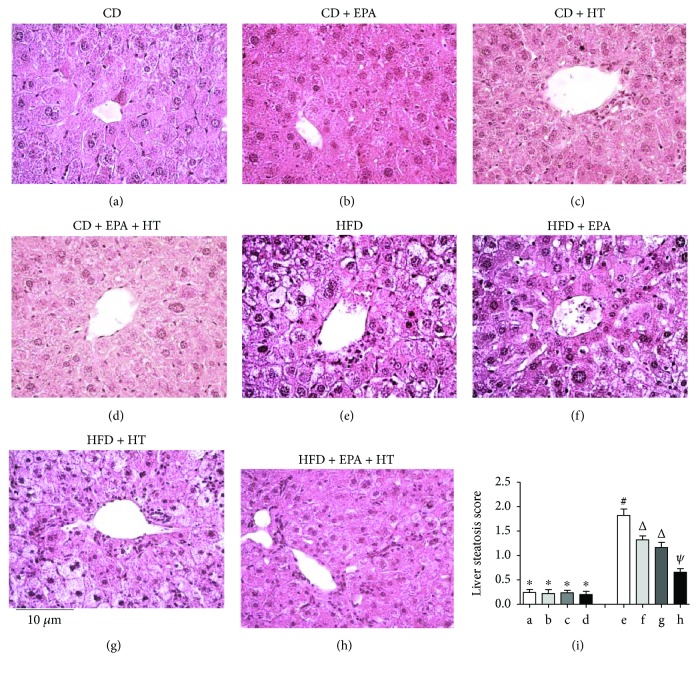
Liver histological assessment in mice subjected to control diet (CD) and high-fat diet (HFD) without and with eicosapentaenoic acid (EPA), hydroxytyrosol (HT), and EPA + HT supplementation. Representative liver sections from animals given (a) CD, (b) CD + EPA, (c) CD + HT, (d) CD + EPA + HT, (e) HFD, (f) HFD + EPA, (g) HFD + HT, and (h) HFD + EPA + HT (haematoxylin-eosin liver sections from 8 animals per experimental group; original magnification ×40). (i) Liver steatosis scores [24] expressed as means ± SEM for 8 animals per experimental group. Groups sharing the same symbol are not significantly different among them according to two-way ANOVA and the Bonferroni posttest (*p* < 0.05). ^∗^^,^^#^^,^^Δ^ and ^ψ^ indicate the significant differences between the experimental groups.

Mice subjected to CD without or with EPA, HT, and EPA + HT supplementation exhibited normal liver histology (Figures 1(a)–1(d)) and showed comparable liver steatosis scores measured according to [35] (Figure 1(i)). HFD for 12 weeks elicited macrovesicular liver steatosis (Figure 1(e)) with 6.5-fold elevation in the steatosis score compared to the CD group (*p* < 0.05), a change that was decreased by 32%, 42%, and 74% by EPA, HT, and EPA + HT supplementation, respectively (*p* < 0.05) (Figure 1(i)).

### 3.3. Liver FA Composition

Total liver SFA, MUFA, and PUFA levels were comparable in all groups subjected to CD (Table 3). However, (i) total SFAs showed 35% increase by HFD over CD values (*p* < 0.05), which was decreased by 25%, 41%, and 22% by EPA, HT, and EPA + HT supplementation (*p* < 0.05); (ii) total MUFAs were not modified in all experimental groups; and (iii) total PUFAs were decreased by 35% by HFD over CD levels (*p* < 0.05), a change that was reduced by 35%, 38%, and 38% by EPA, HT and EPA + HT supplementation, respectively (*p* < 0.05) (Table 3). In relation to total LCPUFAs, mice subjected to CD and EPA or EPA + HT supplementation showed 26% or 34% increases over CD alone (*p* < 0.05), whereas HFD-fed animals exhibited 41% reduction over CD values, an alteration that was reversed by 58%, 38%, and 68% by EPA, HT, and EPA + HT supplementation, respectively (*p* < 0.05) (Table 3). Similarly, HFD induced (i) 35% decrement in n-6 LCPUFA levels (*p* < 0.05) compared to CD values, with EPA, HT, and EPA + HT eliciting 20%, 18%, and 30% recovery, respectively (*p* < 0.05); (ii) 53% reduction in n-3 LCPUFAs (*p* < 0.05) over CD values, whereas EPA, HT, and EPA + HT reached 161%, 74%, and 169% rescue versus HFD alone, with 115% and 130% enhancement by EPA and EPA + HT being found in mice given CD alone; and (iii) 37% increase in the n-6/n-3 LCPUFA ratio (*p* < 0.05), which was lowered by 54%, 32%, and 51% by EPA, HT, and EPA + HT (*p* < 0.05), respectively (Table 3).

### 3.4. Blood Plasma and Liver Oxidative Stress-Related Parameters

**Figure 2:**
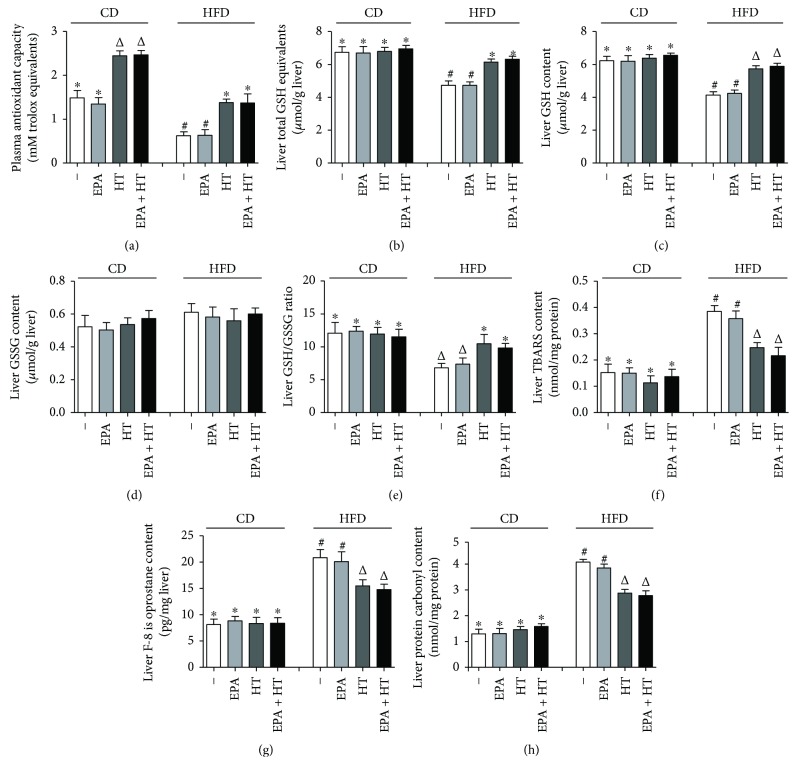
Liver oxidative stress-related parameters in mice subjected to control diet (CD) and high-fat diet (HFD) without (−) and with eicosapentaenoic acid (EPA), hydroxytyrosol (HT), and EPA + HT supplementation. Antioxidant capacity of plasma (a) and contents of total GSH equivalents (b), GSH (c), GSSG (d), GSH/GSSG ratios (e), TBARs (f), F-8 isoprostanes (g), and protein carbonyls (h). Values are means ± SEM for 8 animals per experimental group. Groups sharing the same symbol are not significantly different among them according to two-way ANOVA and the Bonferroni posttest (*p* < 0.05). GSH: reduced glutathione; GSSG: glutathione disulphide; TBARs: thiobarbituric acid reactants. ^∗^^,^^#^ and ^Δ^ indicate the significant differences between the experimental groups.

Mice subjected to CD showed similar values of the antioxidant capacity of plasma when given EPA, which was enhanced by 65% by HT and EPA + HT supplementation (*p* < 0.05); however, HFD elicited 58% reduction in animals without or with EPA treatment that was enhanced by 120% in HT and EPA + HT groups (*p* < 0.05) (Figure 2(a)). Animals given CD without and with supplementations exhibited no significant changes in liver total GSH equivalents (Figure 2(b)), in the levels of GSH (Figure 2(c)) and GSSG (Figure 2(d)), in GSH/GSSG ratios (Figure 2(e)), and in the content of TBARS (Figure 2(f)), F-8 isoprostanes (Figure 2(g)), or protein carbonyls (Figure 2(h)). HFD led to significant decreases in total GSH equivalents (30%), GSH content (34%), and GSH/GSSG ratios (44%), with no alteration in GSSG levels, whereas the contents of TBARS, F-8 isoprostanes, and protein carbonyls were increased by 154%, 157%, and 215%, respectively, over the CD group, changes that were comparable to those found in the HFD + EPA group (Figures 2(b)–2(h)). Compared to the group given HFD alone, HT and EPA + HT recovered to CD values hepatic total GSH equivalents and GSH/GSSG ratios (Figures 2(b) and 2(e)); furthermore, HT and EPA + HT recovered the contents of liver GSH by 77% and 84% (Figure 2(c)), TBARS by 59% and 68% (Figure 2(f)), F-8 isoprostane by 42% and 48% (Figure 2(g)), and protein carbonyls by 43% and 47% (Figure 2(h)), respectively. Under these conditions, antioxidant parameters in plasma (antioxidant capacity) and liver (GSH levels) were significantly correlated (*r* = 0.84; *p* < 0.004), whereas liver GSH contents were inversely associated with those of TBARS (*r* = −0.98; *p* < 0.0001), F-8 isoprostanes (*r* = −0.94; *p* < 0.0002), and protein carbonyls (*r* = −0.93; *p* < 0.0004).

### 3.5. Liver Nrf2 DNA Binding; mRNA Expression of Nrf2, GST, and GGT; and Activity of Enzymes Controlled by Nrf2

**Figure 3:**
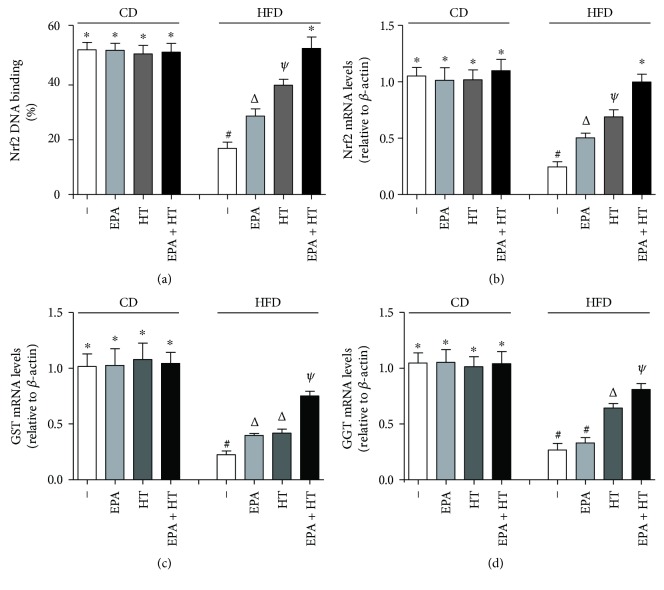
Liver Nrf2 DNA binding (a) and mRNA expression of Nrf2 (b), GST (c), and GGT (d) in mice subjected to control diet (CD) and high-fat diet (HFD) without (−) and with eicosapentaenoic acid (EPA), hydroxytyrosol (HT), and EPA + HT supplementation. Values are means ± SEM for 8 animals per experimental group. Groups sharing the same symbol are not significantly different among them according to two-way ANOVA and the Bonferroni posttest (*p* < 0.05). Nrf2: nuclear factor erythroid 2-related factor 2; GST: glutathione-S-transferase; GGT: *γ*-glutamyl transpeptidase. ^∗^^,^^#^^,^^Δ^ and ^ψ^ indicate the significant differences between the experimental groups.

Mice subjected to CD without and with supplementations revealed comparable values of Nrf2 DNA-binding capacity and in the mRNA expression of Nrf2, GST, and GGT, which were significantly decreased by 68%, 77%, 76%, and 59% over CD values by HFD (*p* < 0.05) (Figures 3(a)–3(d)). HFD-induced decrease in Nrf2 DNA binding exhibited 33% and 64% recovery by EPA and HT, respectively, whereas EPA + HT achieved total recovery (Figure 3(a)); similarly, Nrf2 mRNA levels were recovered by 32%, 55%, and 93% by EPA, HT, and EPA + HT, respectively (Figure 3(b)). Compared to the group given HFD alone, GST mRNA levels were recuperated by 22%, 25%, and 67% by EPA, HT, and EPA + HT, respectively (Figure 3(c)); likewise, EPA, HT, and EPA + HT improved by 8%, 48%, and 70% HFD-induced reduction in GGT mRNA expression (Figure 3(d)).

**Figure 4:**
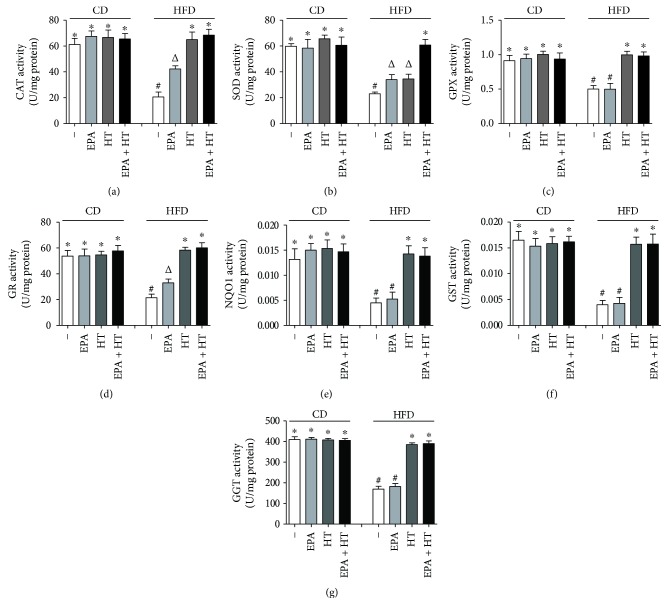
Liver activities of CAT (a), SOD (b), GPX (c), GR (d), NQO1 (e), GST (f), and GGT (g) in mice subjected to control diet (CD) and high-fat diet (HFD) without (−) and with eicosapentaenoic acid (EPA), hydroxytyrosol (HT), and EPA + HT supplementation. Values are means ± SEM for 8 animals per experimental group. Groups sharing the same symbol are not significantly different among them according to two-way ANOVA and the Bonferroni posttest (*p* < 0.05). CAT: catalase; SOD: superoxide dismutase; GPX: glutathione peroxidase; GR: glutathione reductase; NQO1: NADPH-quinone oxidoreductase 1; GST: glutathione-S-transferase; GGT: *γ*-glutamyl transpeptidase. ^∗^^,^^#^ and ^Δ^ indicate the significant differences between the experimental groups.

EPA, HT, and EPA + HT did not elicit significant changes in the activities of the studied antioxidant enzymes in mice fed CD; however, HFD alone decreased those of CAT (66%), SOD (62%), GPX (45%), GR (60%), NQO1 (66%), GST (76%), and GGT (59%) (*p* < 0.05) (Figures 4(a)–4(g)). In HFD-treated animals, (i) EPA did not alter GPX, NQO1, GST, and GGT activities (Figures 4(c), 4(e), 4(f), and 4(g)), but it recovered by 54%, 30%, and 36% those of CAT, SOD, and GR, respectively (Figures 4(a), 4(b), and 4(d)), and (ii) HT improved SOD activity by 31% (Figure 4(b)), whereas (iii) HT and HT + EPA totally recuperated the activities of CAT, GPX, GR, NQO1, GSR, and GGT (Figures 4(a), 4(c)–4(g)).

### 3.6. Correlations

Liver steatosis score was significantly correlated with the content of hepatic TAGs (*r* = 0.94; *p* < 0.0002) and fat content (*r* = 0.97; *p* < 0.0001) and inversely associated with the antioxidant capacity of plasma (*r* = −0.75; *p* < 0.02). The hepatic levels of the antioxidant GSH exhibited an inverse association with the prooxidant parameters, TBARs (*r* = −0.98; *p* < 0.0001), protein carbonyls (*r* = −0.92; *p* < 0.0004), and F-8 isoprostanes (*r* = −0.92; *p* < 0.0012), but revealed a direct correlation with the DNA binding of the redox-sensitive transcription factor Nrf2 (*r* = 0.94; *p* < 0.0004). Moreover, the DNA-binding activity of Nrf2 was significantly associated with the activities of the antioxidant enzymes CAT (*r* = 0.94; *p* < 0.0003), SOD (*r* = 0 0.95; *p* < 0.0001), GPX (*r* = 0.89; *p* < 0.002), GR (*r* = 0.92; *p* < 0.0005), NQO1 (*r* = 0.89; *p* < 0.002), GST (*r* = 0.93; *p* < 0.0005), and GGT (r = 0.89; *p* < 0.002).

## 4. Discussion

Mice subjected to a HFD comprising 60% of the total calories as fat for 12 weeks developed macrovesicular steatosis as evidenced histologically, with a 6.5-fold increase in the steatosis score over the CD values, in agreement with previous studies using either the same dietary protocol [8–10, 18, 34] or alternate procedures [11–13]. Under these conditions, total SFAs were increased by HFD, whereas total PUFAs including n-6 and n-3 LCPUFAs were reduced, changes that may contribute to fatty liver development. HFD-induced liver steatosis score was correlated with the significant enhancements in the contents of liver total fat and TGs, changes that were elicited under conditions of a comparable dietary intake but increased energy intake, and negatively associated with oxidative stress development. The latter phenomenon was characterized by significant alterations in the GSH status lowering the antioxidant potential of the liver with the consequent lipid peroxidation and protein oxidation responses, shown by the elevations in the levels of hepatic TBARs, F-8 isoprostanes, and protein carbonyls, which were inversely correlated with those of GSH. Enhancement of the hepatic oxidative stress status by HFD may be contributed by the drastic decrease in the operation of Nrf2 shown by the lowered Nrf2 DNA-binding capacity and Nrf2 mRNA expression compared to CD, leading to reduced mRNA expression and/or activity of the antioxidant enzymes controlled by Nrf2. Loss of liver Nrf2 activity under sustained oxidative stress conditions triggered by HFD may be related to (i) the prevailing high free-radical level promoting protein oxidation (Figure 2(h)) and inactivation and/or (ii) the increase in the expression of the Nrf2 inhibitor Kelch-like ECH-associated protein 1 (Keap1) which supports continuous proteasomal Nrf2 degradation [36], a mechanism that remains to be evaluated after HFD feeding.

EPA is one of the most important n-3 LCPUFAs due to its roles as (i) DHA precursor, (ii) regulator of hepatic lipid metabolism, which is accomplished by activation of peroxisome proliferator-activated receptor *α* (PPAR-*α*) favouring FA oxidation and downregulation of sterol regulatory element-binding protein 1c (SREBP-1c) reducing de novo lipogenesis [23], and (iii) inhibitor of nuclear factor-*κ*B (NF-*κ*B) limiting inflammatory processes, actions that are shared by DHA [37]. At the dosage of 50 mg/kg/day, EPA supplementation significantly increased the hepatic content of EPA as well as that of DHA, both in mice subjected to CD or HFD. Under these conditions, however, HFD-induced liver steatosis was reduced by only 32% by EPA, without alterations in oxidative stress-related parameters and mild recovery of Nrf2 functioning. This is probably related to transformations that EPA could undergo in the liver, producing metabolic products such as DHA, E-resolvins [38], epoxy-derivatives [39], and/or J_3_ isoprostanes [40], thus limiting the concentration needed for cell signaling.

HT is a polyphenol present in EVOO that has a powerful antioxidant action [41] regulating different signaling pathways associated with the intracellular redox state [42]. This is in agreement with the significant increases in the antioxidant capacity of plasma by HT observed in CD and HFD groups, an effect that is correlated with (i) the total regain towards CD values of the glutathione status (total GSH equivalents and GSH/GSSG ratio), with 77% recovery of GSH levels that were decreased by HFD, and (ii) the partial recovery (42% to 59%) of parameters related to free radical-induced lipid peroxidation (TBARs and F-8 isoprostanes) and protein carbonylation that were enhanced by HFD, indexes negatively correlated with GSH levels. Under these conditions, reduction in HFD-induced liver oxidative stress status by HT is associated with enhancement (55%) in the mRNA expression of Nrf2, with 64% regain in its DNA-binding capacity, responses that may contribute to an increase in the antioxidant potential of the liver [24]. This contention is supported by the total recovery of the activity of the enzymes controlled by Nrf2 that were decreased by HFD, namely, CAT, GPX, GR, NQO1, GST, and GGT, with the partial regain in that of SOD observed after HT supplementation. An additional effect elicited by HT favouring the antioxidant potential of the liver under HFD-induced oxidative stress conditions is the enhancement in the content of total LCPUFAs including that of total n-3 LCPUFAs, possibly by decreasing their oxidative deterioration [43]. This effect of HT is likely to promote PPAR-*α* activation increasing the FA oxidation capacity of the liver with concomitant SREBP-1c downregulation reducing de novo lipogenesis [43], thus in agreement with the 42% reduction found in HFD-induced steatosis score that correlated with the decreases in the fat and TG contents.

HFD-fed mice subjected to combined supplementation with EPA + HT showed a significant greater and additive antisteatotic effect (74% reduction) compared to that elicited by the separate HT (42% diminution) and EPA (32% decrease) treatments, with hepatic fat and TG contents being significantly lower than those induced by HFD alone. However, the effects of EPA + HT along with HFD feeding were either (i) similar to those achieved by HT alone, namely, total regain in n-3 LCPUFA levels, plasma antioxidant capacity, glutathione status, and CAT, GPX, GE, NQO1, GST, and GGT activities, or (ii) showed partial to total recovery transition of Nrf2 functioning and SOD activity, when values for HT alone and EPA + HT are compared. These data indicate that the antisteatotic and antioxidant effects produced by combined EPA + HT supplementation in HFD feeding are mainly due to the HT component, with EPA having a limited contribution at the dosages employed. This conclusion points to antioxidation as a major mechanism underlying attenuation of HFD-induced steatosis by natural products. In agreement with this proposal, mitigation of HFD-induced liver deleterious effects is also attained by both (i) an EVOO type having the highest antioxidant content (859 mg polyphenols/kg; antioxidant capacity of 7156 *μ*mol eq. Trolox/L) compared to those having polyphenol levels of 116 or 407 mg/kg and antioxidant capacities of 3378 or 4841 *μ*mol eq. Trolox/L, respectively [34], and (ii) a regular rosa mosqueta oil (RMO) containing *α*- and *γ*-tocopherols compared to a RMO type devoid of tocopherols [44], findings that establish a threshold for the content of antioxidant components of natural products to achieve beneficial effects. In addition to the antioxidant effect of natural products in fatty liver, reduction of HFD-induced liver steatosis by EPA, HT, and EPA + HT supplementation may result from a direct activation of hepatic lipases such as patatin-like phospholipase domain-containing protein 3 (PNPLA3) which hydrolyses acylglycerols including TGs [45], an aspect that remains to be elucidated in the present model.

## 5. Conclusion

Data presented show that combined EPA + HT supplementation in mice significantly attenuates HFD-induced hepatic steatosis, an effect that mainly relies on HT with a limited contribution of EPA. Under these conditions, the antisteatotic effect of HT is associated with the enhancement in the antioxidant potential of the liver, which partially recovers n-3 LCPUFA levels thus favouring FA oxidation through PPAR-*α* upregulation [35], while limiting de novo lipogenesis via SREBP-1c downregulation [43]. Additional mechanisms of HT action include (i) prevention of HFD-induced reduction in the desaturation capacity of the liver, with recovery in the activity of *Δ*5 and *Δ*6 desaturases promoting n-3 LCPUFA repletion [43]; (ii) reduction in the oxidative stress-dependent liver protein carbonylation triggered by HFD, thus decreasing the lipogenic response associated with the endoplasmic reticulum stress (ERS) developed [46, 47], an effect that may be contributed by normalization of SFA levels increased by HFD, FAs that also trigger ERS [48]; and (iii) amelioration of drug-induced cardiotoxicity involving oxidative stress and mitochondrial dysfunction [49], which suggest enhancement in electron transport chain capacity and FA oxidation potential [21]. These considerations and the previous suggestions concerning the adequacy of combination therapies [14–20] reinforce the impact of dietary interventions including safety components addressing oxidative stress as a central mechanism underlying liver steatosis in obesity in particular and other noncommunicable diseases in general.

## Figures and Tables

**Table 1:** Gene-specific primer sequences used in the study.

mRNA	Forward primer	Reverse primer
*Nrf2*	AAGCTTTCAACCCGAAGCAC	TTTCCGAGTCACTGAACCCA
*Gst*	TGCAGACCAAAGCCATTCTC	ACGGTTCCTGGTTTGTTCCT
*Ggt*	ATGTGGACACCCGATGCAGTATT	TGTCTTGCTTGTAGTCAGGATGGTTT
*β-Actin*	ACTGCCGCATCCTCTTCCTC	CTCCTGCTTGCTGATCCACATC

Sequences are listed in the 5′ → 3′ direction. *Nrf2*: nuclear factor erythroid 2-related factor 2; *Gst*: glutathione-S-transferase; *Ggt*: *γ*-glutamyl transpeptidase.

**Table 2:** General and hepatic parameters in control mice (CD) and high-fat diet (HFD) animals subjected to EPA, HT, and EPA plus HT. Values are presented as means 10 ± 8 mice per experimental group. Significant differences between the groups are indicated by the letter identifying each group (*p* < 0.05), by two-way ANOVA and Bonferroni posttest

	Control diet (CD)				High-fat diet (HFD)			
	Saline	EPA	HT	EPA + HT	Saline	EPA	HT	EPA + HT
General parameters	(a)	(b)	(c)	(d)	(e)	(f)	(g)	(h)
Initial body weight (g)	14.71 ± 1.02	14.56 ± 1.11	14.38 ± 0.99	14.51 ± 0.91	14.26 ± 0.74	14.77 ± 1.02	14.31 ± 0.86	14.56 ± 0.97
Final body weight (g)	26.67 ± 3.51^e,g^	25.47 ± 4.24^e,g^	28.79 ± 1.76 ^e,g^	26.96 ± 4.44	37.15 ± 5.01^a,b,c,d^	33.13 ± 3.56^c^	36.02 ± 3.15^a,b,c,d^	30.00 ± 6.60^e,g^
Total body weight increment (g)	11.95 ± 3.74^e^	10.91 ± 4.44^g^	14.42 ± 1.88^h^	12.45 ± 4.02	22.89 ± 5.40^a,b,c,d^	18.36 ± 2.99^c^	21.71 ± 2.91^a,b,c,d^	15.44 ± 6.11^e,g^
Liver weight (g)	1.25 ± 0.23^h^	1.04 ± 0.18	1.17 ± 0.14	1.07 ± 0.19	1.12 ± 0.11	1.19 ± 0.23^h^	1.23 ± 0.16^h^	1.03 ± 0.77^a,fg^
Food and energy intake								
Dietary intake (g/day)	5.08 ± 0.44	5.24 ± 0.21	5.18 ± 0.32	4.99 ± 0.69	5.29 ± 0.37	5.18 ± 0.30	5.15 ± 0.41	5.24 ± 0.38
Energy intake (kcal/day)	19.59 ± 1.28^e,f,g,h^	20.18 ± 1.42^e,f,g,h^	19.95 ± 1.87^e,f,g,h^	19.20 ± 1.36^e,f,g,h^	27.74 ± 2.31^a,b,c,d^	27.15 ± 2.24^a,b,c,d^	26.99 ± 2.19^a,b,c,d^	27.5 ± 2.04^a,b,c,d^
Liver parameters								
AST (U/L)	152.67 ± 15.02	143.00 ± 15.11	142.67 ± 12.31	145.33 ± 11.44	151.83 ± 16.65	150.33 ± 9.38	152.50 ± 9.07	151.00 ± 14.87
ALT (U/L)	62.50 ± 6.63	64.00 ± 10.41	63.33 ± 7.63	59.33 ± 7.63	70.83 ± 6.69	69.83 ± 7.67	69.33 ± 8.67	64.67 ± 10.48
Hepatic fat g/100 g liver)	4.98 ± 0.88^e,f,g,h^	3.81 ± 0.68^e,f,g,h^	4.91 ± 1.31^e,f,g,h^	3.13 ± 0.57^e,f,g,h^	14.59 ± 1.69^a,b,c,d,h^	10.12 ± 1.06^a,b,c,d,h^	11.26 ± 1.11^a,b,c,d,h^	8.49 ± 1.05^a,b,c,d,e,f,g^
Hepatic TAG (mg/g liver)	33.05 ± 7.19^e,f,g,h^	30.38 ± 5.52^e,f,g,h^	29.48 ± 3.95^e,f,g,h^	27.74 ± 5.09^e,f,g,h^	102.47 ± 9.47^a,b,c,d,h^	56.70 ± 5.74^a,b,c,d,^	77.89 ± 7.21^a,b,c,d,h^	49.94 ± 1.45^a,b,c,d,e,f,g^

**Table 3:** Total hepatic fatty acid profile in control mice (CD) and high-fat diet (HFD) animals subjected to EPA, HT, and EPA plus HT. Values are presented as mean % of fatty acid methyl esters (FAME) 10 ± 8 mice per experimental group. Significant differences between the groups are indicated by the letter identifying each group (*p* < 0.05), by two-way ANOVA and Bonferroni posttest. Saturated fatty acids (SFA) are 10 : 0, 12 : 0, 14 : 0, 16 : 0, 18 : 0, 20 : 0, 22 : 0, and 24 : 0. Monounsaturated control mice (CD) and high-fat diet (HFD) animals subjected to EPA, HT, and EPA plus HT d fatty acids (MUFA) are 14 : 1, 16 : 1, 18 : 1, 20 : 1 n-9, and 22 : 1 n-9 to 24 : 1. Polyunsaturated fatty acids (PUFAs) are 18 : 2 n-6 (linoleic acid (LA)), 18 : 3 n-6, 18 : 3 n-3 (*α*-linolenic acid (ALA)), 20 : 2 n-6, 20 : 3 n-6, 20 : 3 n-3, 20 : 4 n-6 (arachidonic acid (ARA)), 20 : 5 n-3, 22 : 5 n-3 (docosapentaenoic acid (DPA)), and 22 : 6 n-3. Long-chain polyunsaturated fatty acids (LCPUFA) are 20 : 2 n-6: 20 : 3 n-6: 20 : 4 n-6, 20 : 5 n-3 (eicosapentaenoic acid (EPA)), 22: 5 n-3, and 22 : 6 n-3 (docosahexaenoic acid (DHA)).

	Fatty acid composition (g per 100 g FAME)							
	Control diet (CD)				High-fat diet (HFD)			
	Saline	EPA	HT	EPA + HT	Saline	EPA	HT	EPA + HT
Most relevant fatty acids	(a)	(b)	(c)	(d)	(e)	(g)	(f)	(h)
16 : 00	34.6 ± 3.6^e^	33.8 ± 3.5^e^	31.2 ± 3.8^e^	34.7 ± 3.3^e^	47.9 ± 4.1^a,b,c,d,f,g,h^	38.7 ± 2.9^e^	36.9 ± 3.1^e^	37.2 ± 3.2^e^
18 : 01	25.3 ± 2.5	24.3 ± 2.7	26.5 ± 2.2	23.9 ± 2.6	26.8 ± 2.8	23.5 ± 2.3	24.4 ± 2.9	24.7 ± 2.6
18 : 2, n-6 (AL)	10.4 ± 1.3	9.98 ± 1.2	10.2 ± 1.1	9.75 ± 1.1	10.9 ± 1.3	9.71 ± 1.0	10.5 ± 1.4	10.1 ± 1.2
18 : 3, n-3 (ALA)	1.15 ± 0.1^e^	1.14 ± 0.06^e^	1.10 ± 0.1^e^	1.12 ± 0.05^e^	0.81 ± 0.03^a,b,c,d,f,g,h^	1.09 ± 0.1^e^	1.11 ± 0.1^e^	1.07 ± 0.2^e^
20 : 4, n-6 (AA)	9.93 ± 1.5^e^	8.64 ± 1.5^e^	10.5 ± 1.7^e^	8.98 ± 1.6^e^	6.61 ± 0.6^a,b,f,h^	7.85 ± 1.1	7.84 ± 1.4	8.56 ± 1.2^e^
20 : 5, n-3 (EPA)	1.03 ± 0.1^b,d,e,g,h^	3.98 ± 0.5^a,c,e,f,g,h^	1.12 ± 0.1^b,d,e,g,h^	4.16 ± 0.6^a,c,e,f,g,h^	0.34 ± 0.05^a,b,c,d,f,g,h^	2.14 ± 0.2^a,b,c,d,e,f^	0.99 ± 0.1^c,d,e,g,h^	2.36 ± 0.2^a,b,c,d,e,f^
22 : 6, n-3 (DHA)	3.96 ± 0.3^b,d,e^	6.94 ± 0.5^c,d,e^	4.12 ± 0.2^b,d,e^	7.12 ± 0.7^b,d,e^	1.97 ± 0.1^a,b,c,d,f,g,h^	3.85 ± 0.4^b,d,e^	3.16 ± 0.3^c,d,e^	4.03 ± 0.4^b,d,e^
Total SFA	37.8 ± 3.4^e^	38.9 ± 3.9^e^	35.4 ± 3.3^e^	37.4 ± 3.4^e^	51.2 ± 4.9^a,b,c,d,f,g,h^	38.6 ± 3.5^e^	30.4 ± 3.2^e^	39.9 ± 3.4^e^
Total MUFA	29.5 ± 2.2	27.9 ± 2.8	31.3 ± 2.9	28.8 ± 2.6	27.7 ± 2.5	32.9 ± 3.0	30.5 ± 3.1	30.9 ± 3.1
Total PUFA	32.7 ± 2.9^e^	33.2 ± 3.2^e^	33.3 ± 3.5^e^	33.8 ± 3.5^e^	21.1 ± 1.9^a,b,c,d,f,g,h^	28.5 ± 2.5^e^	29.1 ± 2.2^e^	29.2 ± 2.5^e^
Total LCPUFA	15.6 ± 1.2^b,d,e,f^	19.8 ± 1.6^a,c,e,f,g,h^	16.2 ± 1.4^b,d,e,f^	20.9 ± 2.1^a,c,e,f,g,h^	9.13 ± 0.5^a,b,c,d,f,g,h^	14.4 ± 1.1^b,d,e,f^	12.1 ± 0.7^a,b,c,d,e,g,h^	15.3 ± 1.3^b,d,e,f^
Total n-6 LCPUFA	10.4 ± 0.8^b,e,f,g,h^	8.80 ± 0.7^a,c,e^	10.8 ± 1.0^b,e,f,g,h^	9.10 ± 0.9^e,f,^	6.71 ± 0.5^a,b,c,d,g,h^	8.08 ± 0.6^a,c,e^	7.90 ± 0.6^a,c,d,e^	8.78 ± 0.7^a,c,e^
Total n-3 LCPUFA	5.12 ± 0.2^b,d,e,f,g,h^	11.0 ± 0.4^a,c,e,f,g,h^	5.33 ± 0.2^b,d,e,f,g,h^	11.8 ± 0.4^a,c,e,f,g,h^	2.42 ± 0.2^a,b,c,d,f,g,h^	6.32 ± 0.5^a,b,c,d,e,f^	4.20 ± 0.3^a,b,c,d,e,g,h^	6.52 ± 0.5^a,b,c,d,e,f^
n-6/n-3 LCPUFA ratio	2.03 ± 0.3^b,d,g,h^	0.80 ± 0.05^a,c,e,f,g,h^	2.03 ± 0.2^b,d,g,h^	0.77 ± 0.04^a,c,e,f,g,h^	2.77 ± 0.3^b,d,g,h^	1.28 ± 0.1^a,b,c,d,e,f^	1.88 ± 0.2^c,d,f,g,h^	1.35 ± 0.1^a,b,c,d,e,f^

## Data Availability

The data used to support the findings of this study are available from the corresponding author upon request.
